# 3′ *Tth* Endonuclease Cleavage Polymerase Chain Reaction (3TEC-PCR) Technology for Single-Base-Specific Multiplex Pathogen Detection using a Two-Oligonucleotide System

**DOI:** 10.3390/ijms22116061

**Published:** 2021-06-04

**Authors:** Owen Higgins, Terry J. Smith

**Affiliations:** 1 Molecular Diagnostics Research Group, School of Natural Sciences, National University of Ireland, Galway, Ireland; terry.smith@nuigalway.ie; 2 Centre for One Health, Ryan Institute, National University of Ireland, Galway, Ireland

**Keywords:** nucleic acid diagnostics, polymerase chain reaction, multiplex detection, single-nucleotide polymorphism, bacterial meningitis

## Abstract

Polymerase chain reaction (PCR) is the standard in nucleic acid amplification technology for infectious disease pathogen detection and has been the primary diagnostic tool employed during the global COVID-19 pandemic. Various PCR technology adaptations, typically using two-oligonucleotide dye-binding methods or three-oligonucleotide hydrolysis probe systems, enable real-time multiplex target detection or single-base specificity for the identification of single-nucleotide polymorphisms (SNPs). A small number of two-oligonucleotide PCR systems facilitating both multiplex detection and SNP identification have been reported; however, these methods often have limitations in terms of target specificity, production of variable or false-positive results, and the requirement for extensive optimisation or post-amplification analysis. This study introduces 3′ *Tth* endonuclease cleavage PCR (3TEC-PCR), a two-oligonucleotide PCR system incorporating a modified primer/probe and a thermostable cleavage enzyme, *Tth* endonuclease IV, for real-time multiplex detection and SNP identification. Complete analytical specificity, low limits of detection, single-base specificity, and simultaneous multiple target detection have been demonstrated in this study using 3TEC-PCR to identify bacterial meningitis associated pathogens. This is the first report of a two-oligonucleotide, real-time multiplex PCR technology with single-base specificity using *Tth* endonuclease IV.

## 1. Introduction

Nucleic acids, ribonucleic acid (RNA) and deoxyribonucleic acid (DNA), are hereditary macromolecule biopolymers composed of nucleotide subunits. Each nucleotide contains a five-carbon sugar coordinated with a 5′ phosphate group, a 3′ hydroxyl group, and a 1′ pyrimidine or purine nitrogenous base. Strands of nucleotides have a 5′ phosphate group at one end and a 3′ hydroxyl group at the other, to which new nucleotides are added, resulting in 5′ to 3′ sense or 3′ to 5′ antisense directionality. DNA consists of two antiparallel nucleotide strands in a double helix structure joined together by hydrogen bonds that link complementary nitrogenous bases: guanine with cytosine and adenine with thymine [1,2]. This complementary DNA base pairing is highly specific and utilised in molecular diagnostics for infectious disease pathogen detection [3]. Nucleic acid amplification diagnostics, such as polymerase chain reaction (PCR), are used to target pathogen specific genomic nucleotide sequences and amplify these biomarkers to detectable levels [4]. Although PCR was first conceived in the 1980s [5,6,7,8], it remains the most commonly used nucleic acid amplification technology and has been the primary diagnostic tool of choice during the global COVID-19 pandemic [9,10,11].

PCR is a temperature-mediated technology that mimics aspects of DNA replication by rapidly cycling high-temperature DNA denaturing phases with specific lower-temperature annealing phases. This process causes separation of the target DNA to be amplified, enabling sense and antisense, or forward and reverse, single-stranded oligonucleotide primers to complement bind at opposing ends of a short nucleotide sequence located in the biomarker target. The free 3′ hydroxyl group of each hybridised primer is targeted by a thermostable DNA polymerase leading to the addition of nucleotide subunits, generating new DNA strands. Each denaturation and annealing temperature cycle doubles the biomarker target, leading to exponential amplification that can be monitored in real-time, typically using thermocycling fluorometers in combination with hydrolysis probes or DNA-binding dyes. Hydrolysis probes are single-stranded oligonucleotides, with a 5′ fluorophore and 3′ quencher, located between PCR primers and designed with higher melting temperatures to ensure earlier target hybridisation. The 3′ quencher absorbs fluorophore fluorescence and acts as an extension blocker preventing non-specific signal generation. Extension from the flanking primers during each PCR cycle dissociates the hydrolysis probe fluorophore and quencher producing fluorescence which is relative to the amplified product, enabling both target detection and quantification [6,12]. Real-time PCR monitoring using the two-oligonucleotide system of a forward and reverse primer with nucleic acid dyes facilitates simpler assay design and lower cost compared to the three-oligonucleotide hydrolysis probe system [13]. However, the dye-binding two-oligonucleotide system is less specific and more prone to primer-dimer formation or non-target amplification, leading to false-positive results [14].

Various PCR applications or performance properties such as multiplex target detection and single-base specificity are essential for effective infectious disease diagnostics. Multiplex PCR involves the simultaneous detection of multiple targets in a single reaction, enabling reduced analysis time and reagent cost, conservation of clinical specimen, and incorporation of assay validating internal controls [15,16]. Typically, multiplex PCR is achieved using the three-oligonucleotide hydrolysis probe system with differentially coloured fluorophores and multichannel thermocycling fluorometers. The dye-binding two-oligonucleotide PCR system also enables multiplex detection; however, this approach requires post-amplification melt analysis and is more prone to assay cross-reactivity [12,17]. Single-base specificity in PCR is an important performance property as it can enable effective differentiation between closely related targets as well as the identification of specific single-nucleotide polymorphisms (SNPs). SNPs are genomic point mutations or single nucleotide differences found in at least 1% of a population. Particular SNPs can be associated with various diseases, pathogenic microorganisms, or antimicrobial resistance, and as a result are commonly targeted in molecular diagnostics. SNP variations at a particular genome location are referred to as alleles, with the most commonly found variation in a population known as the wild-type allele and alterations from this referred to as mutant alleles [18,19,20,21]. SNP detection using single-base-specific PCR has been demonstrated with both allele-biased amplification [22,23] and allele-biased signal generation methods [24,25]. Allele-biased amplification, such as allele-specific PCR, biases amplification of a particular allele by locating SNP mismatches at primer 3′-ends to inhibit polymerisation and enable differentiation between variant alleles. This approach, however, produces variable results and generally requires extensive optimisation to successfully differentiate targets [26]. Another major limitation to this method is that once a non-specific priming event of the SNP template occurs, incorporating primers into the amplicon, no further target discrimination can be achieved. Allele-biased signal generation methods, such as the three-oligonucleotide hydrolysis probe system, differentiate between unbiased allele amplification by designing probes to coordinate with a particular allele SNP. For effective SNP differentiation using this method, shorter hydrolysis probes are used, requiring the incorporation of modified bases such as locked nucleic acids (LNAs) to maintain appropriate probe melting temperature values [27,28].

**Figure 1:**
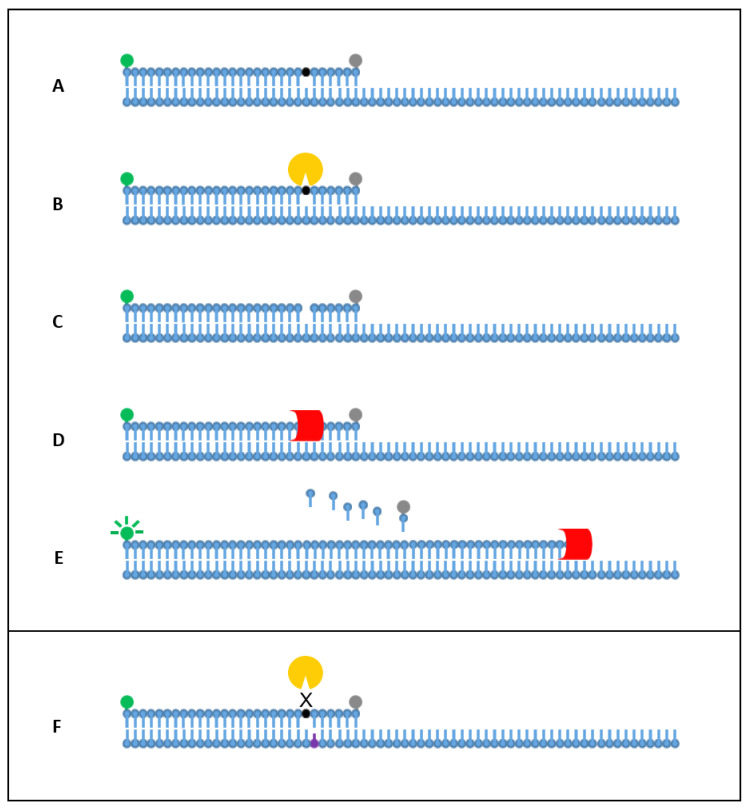
3TEC-PCR detection mechanism. (**A**) The 3TEC-PCR primer/probe, containing a 5′ fluorophore (green), an internal abasic site (black), and a 3′ quencher (grey), hybridises to its complementary target during PCR annealing. The quencher, coordinated to the 3′-end hydroxyl group, absorbs fluorophore signal and prevents DNA polymerisation. (**B**) After target hybridisation, the abasic site (black) is in duplex form leading to *Tth* endonuclease IV (yellow) cleavage of the phosphodiester bond. (**C**) Cleavage of the abasic site leaves a single nucleotide gap with a free 3’ hydroxyl group. (**D**) DNA polymerase (red) coordinates to the free hydroxyl group, initiating strand extension. (**E**) Exonuclease activity of the polymerase enzyme dissociates nucleotides downstream of the abasic site, leading to fluorescence production from fluorophore and quencher separation. (**F**) Presence of a single-nucleotide polymorphism (purple) on the 3′-side of the abasic site prevents complete duplex structure formation, thus inhibiting cleavage and preventing fluorescence production.

This article introduces 3′ *Tth* endonuclease cleavage PCR (3TEC-PCR) technology, a novel two-oligonucleotide PCR system that enables real-time multiplex target detection with single-base specificity. The 3TEC-PCR method uses standard PCR conditions with a modified primer/probe and a thermostable endonuclease, *Tth* endonuclease IV. The modified primer/probe incorporates a 5′ fluorophore, an internal abasic site and a 3′ quencher (Figure 1). In single-stranded form, the modified primer/probe is quenched and blocked from polymerase extension by the 3′ quencher. In target-bound duplex form, the modified primer/probe abasic site is cleaved by the *Tth* endonuclease IV enzyme, enabling extension and fluorescence production via dissociation of the fluorophore and quencher. The presence of an SNP immediately 3′ of the abasic site causes cleavage inhibition, which facilitates the 3TEC-PCR single-base specificity. In this study, we have demonstrated 3TEC-PCR real-time detection, analytical specificity and sensitivity, SNP differentiation, and multiplex detection using bacterial meningitis pathogens *Haemophilus influenzae*, *Neisseria meningitidis,* and *Streptococcus pneumoniae* as model target organisms.

## 2. Results

### 2.1. Singleplex Detection

**Figure 2:**
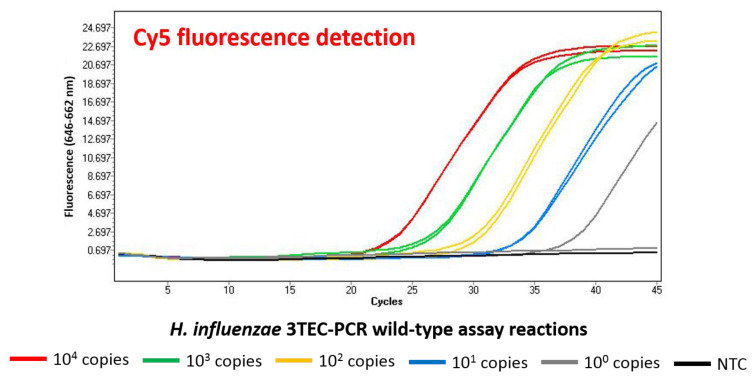
3TEC-PCR singleplex detection. The *H. influenzae* 3TEC-PCR wild-type assay was tested with 10-fold serially diluted *H. influenzae* type strain genomic DNA, with a no-template control reaction performed in parallel. Resulting amplification fluorescence was recorded with the LightCycler 480 Instrument II using the Cy5 fluorescence detection channel. Genome copy concentrations tested at 10^4^ (red), 10^3^ (green), 10^2^ (yellow), and 10^1^ (blue) were successfully detected in duplicate, with one of the 10^0^ (grey) duplicates detected. No signal was observed in the negative control reaction (black).

The *H. influenzae* 3TEC-PCR wild-type assay successfully detected each duplicate of the 10-fold serially diluted type strain *H. influenzae* DNA concentrations tested at 10^4^, 10^3^, 10^2^, and 10^1^ genome copies per reaction, and detected one of the duplicates tested at 10^0^ genome copies (Figure 2). The resulting amplification curves produced approximate average Ct values of 23.5, 27, 30.5, and 34 for the 10^4^, 10^3^, 10^2^, and 10^1^ genome copy number concentrations, respectively, and produced a Ct value of 38 for one of the 10^0^ genome copy duplicates. The negative control reaction performed appropriately as no fluorescence signal was observed.

### 2.2. Analytical Specificity and Sensitivity

Analytical specificity testing of the *H. influenzae* 3TEC-PCR wild-type assay resulted in the accurate detection of all 10 of the inclusivity panel *H. influenzae* references strains analysed. No detection was observed for all 30 of the exclusivity panel closely related *Haemophilus* reference strains tested or the *N. meningitidis* and *S. pneumoniae* type strains (Table S1). This result indicated complete analytical specificity for the *H. influenzae* 3TEC-PCR wild-type assay.

Analytical sensitivity testing of the *H. influenzae* 3TEC-PCR wild-type assay using serially diluted type strain *H. influenzae* genomic DNA resulted in all 12 replicates tested at 32, 16, and 8 genome copy concentrations being successfully detected. No detection was observed for one of the 4 genome copy replicates, two of the 2 genome copy replicates and five of the 1 genome copy replicates tested. Probit analysis performed on this resulting data indicated that the *H. influenzae* 3TEC-PCR wild-type assay produced an LOD with 95% confidence of 4.1 genome copies per reaction (Table S3).

### 2.3. Allele-Specific Detection

**Figure 3:**
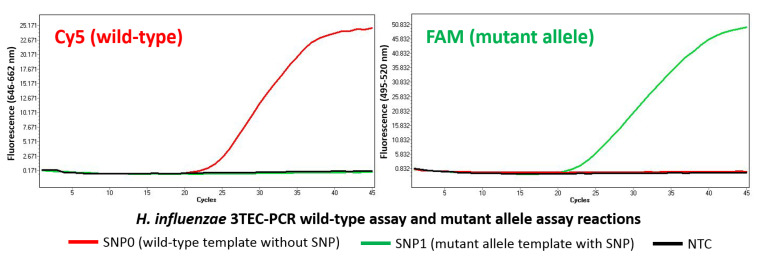
3TEC-PCR allele-specific detection. The *H. influenzae* 3TEC-PCR wild-type assay and mutant allele assay were both tested using synthetic DNA templates, SNP0 and SNP1, at 10^4^ genome copies with no template control reactions carried out in parallel. SNP0 is a sectional copy of the *H. influenzae fucK* gene, without SNPs, acting as a wild-type template. SNP1 is a copy of the SNP0 template, with an SNP in the *H. influenzae* wild-type forward primer/probe target region, acting as a mutant allele template. Resulting amplification fluorescence was recorded with the LightCycler 480 Instrument II using the Cy5 (wild-type) and FAM (mutant allele) fluorescence-detection channels. The SNP0 template (red) was only detected in the Cy5 channel and the SNP1 template (green) was only detected in the FAM channel, indicating single-base specificity and accurate allele-specific detection with both assays. The negative control reactions performed successfully as no signal generation was observed (black).

The *H. influenzae* 3TEC-PCR wild-type assay and mutant allele assay both successfully exhibited single-base specificity (Figure 3). The *H. influenzae* 3TEC-PCR wild-type assay accurately detected the SNP0 wild-type template, a 500 bp sectional copy of the *H. influenzae fucK* gene biomarker target, at 10^4^ copies per reaction with a resulting Ct value of 23.5. In parallel, the wild-type assay did not detect the SNP1 mutant allele template, a copy of the SNP0 template with an SNP in the *H. influenzae* wild-type forward primer/probe target region. The *H. influenzae* 3TEC-PCR mutant allele assay correctly detected the SNP1 mutant allele template at 10^4^ copies per reaction with a resulting Ct value of 23 and did not detect the SNP0 wild-type template. The negative control reactions for both assays performed as expected with no signal detection observed.

### 2.4. Multiplex Detection

**Figure 4:**
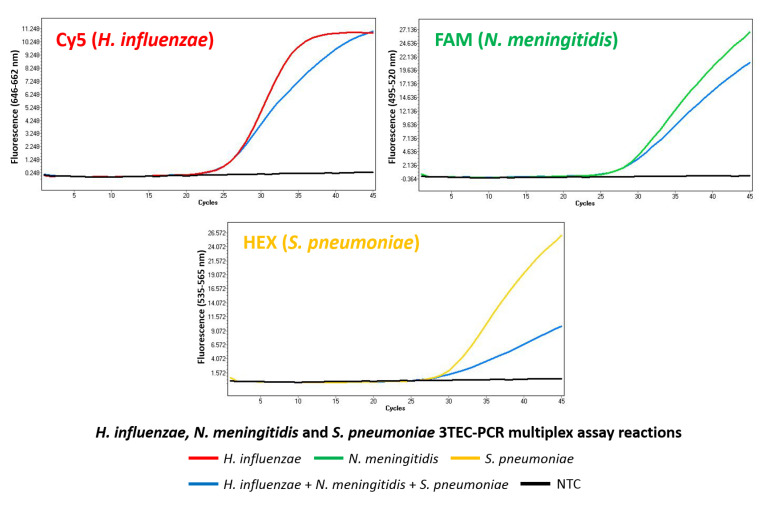
3TEC-PCR multiplex detection. The *H. influenzae*, *N. meningitidis*, and *S. pneumoniae* 3TEC-PCR multiplex assay was challenged with type strain genomic DNA from all three bacterial targets at 10^4^ genome copy concentrations, in separate reactions and in one combined reaction. Resulting amplification fluorescence was recorded with the LightCycler 480 Instrument II using the Cy5, FAM, and HEX fluorescence detection channels. Each bacterial target tested in separate reactions was successfully detected in the appropriate corresponding detection channel: *H. influenzae* in Cy5 (red), *N. meningitidis* in FAM (green), and *S. pneumoniae* in HEX (yellow), with no non-specific detection observed. The successful simultaneous detection of all three bacterial targets in a single reaction was also observed (blue). The negative control reaction performed successfully as no signal generation was observed (black).

The *H. influenzae*, *N. meningitidis,* and *S. pneumoniae* 3TEC-PCR multiplex assay accurately identified all three bacterial targets tested at 10^4^ genome copies, in separate reactions and in one combined reaction (Figure 4). All bacterial targets were detected in appropriate corresponding fluorescence-detection channels: *H. influenzae* in Cy5, *N. meningitidis* in FAM, and *S. pneumoniae* in HEX, with no non-specific target detection or channel-to-channel fluorescence cross-talk observed. Each bacterial target produced the same approximate Ct value when tested separately or when tested in combination with the other two bacterial targets in the same reaction: 26 for *H. influenzae*, 28 for *N. meningitidis*, and 29 for *S. pneumoniae*. There was no amplification fluorescence signal observed in the negative control reaction performed in parallel, indicating no non-specific detection or unwanted interaction between the 3TEC-PCR oligonucleotide sets.

## 3. Discussion

Real-time PCR, typically performed using a two-oligonucleotide dye-binding method or a three-oligonucleotide hydrolysis probe system, is the most widely used nucleic acid diagnostic approach for the identification of infectious disease pathogens [9,12]. Multiplex target detection and single-base specificity are essential diagnostic assay performance properties for effective PCR application. Multiplex PCR enables simultaneous detection of multiple targets in a single reaction, reducing assay costs and facilitating internal control incorporation [15,16]. Single-base specificity enables highly specific PCR and the identification of single-nucleotide polymorphisms (SNPs) using either allele biased amplification or allele biased signal generation methods [22,24]. In this study, we introduce a novel two-oligonucleotide PCR system that enables real-time multiplex target detection with single-base specificity, 3′ *Tth* endonuclease cleavage PCR (3TEC-PCR). Bacterial-meningitis-associated pathogens, *H. influenzae*, *N. meningitidis*, and *S. pneumoniae*, were used to demonstrate the real-time detection, analytical specificity and sensitivity, SNP differentiation, and multiplex detection capabilities of the 3TEC-PCR method.

Singleplex 3TEC-PCR real-time target detection was exemplified in this study using the *H. influenzae* 3TEC-PCR wild-type assay (Figure 2). Standard real-time PCR typically produces sigmoidal shaped fluorescence amplification curves with lag, log, and stationary phases, representing target concentration as a function of the PCR cycle on a linear scale [29]. Efficient real-time PCR reactions are exponential and cause the doubling of target product at each cycle, typically producing Ct value differences of approximately 3.3 cycles between 10-fold serially diluted target concentrations [30]. Ct values higher than 40 cycles, indicating low-level detection, are usually disregarded or considered questionable [31]. The resulting amplification curves from the *H. influenzae* 3TEC-PCR wild-type assay (Figure 2) are sigmoidal in shape with an approximate Ct value difference of 3.5–4 between each of the 10-fold serially diluted concentrations of *H. influenzae* DNA tested, typical of standard real-time PCR. Each DNA concentration tested, apart from the single genome copy concentration, was successfully detected in duplicate, producing similar amplification curves for each replicate. This result illustrates the reproducibility of the 3TEC-PCR assay, as well as sensitive low-level detection considering the single genome copy detection was observed before the 40-cycle threshold. As signal generation was not observed in the negative control reaction, this indicates that no non-specific interactions occurred using the 3TEC-PCR method. Complete analytical specificity was displayed using the 3TEC-PCR method as only the inclusivity panel strains were detected during specificity testing of the *H. influenzae* 3TEC-PCR wild-type assay (Table S1). Additionally, sensitive low-level detection using the 3TEC-PCR method was highlighted, as the *H. influenzae* 3TEC-PCR wild-type assay produced an LOD with 95% confidence of 4.1 genome copies per reaction (Table S3). To obtain this value, 12 replicates of serially diluted *H. influenzae* genomic DNA concentrations were tested. The minimum number of replicates required to give statistically valid LOD results is six; however, testing a higher number of replicates increases the confidence of the sensitivity testing [32]. The lowest target concentration at which 95% of positive samples are detected is referred to as the LOD, and the theoretical LOD for PCR is limited to 3 genome copies per reaction according to Poisson distribution [31].

The single-base specificity of 3TEC-PCR was demonstrated using the *H. influenzae* 3TEC-PCR wild-type and mutant allele assays in combination with synthetic DNA templates, SNP0 and SNP1 (Figure 3). Each assay only detected the complementary SNP-free target and did not detect the non-complementary SNP-containing target. This result indicates that an SNP present on the 3′ side of the forward primer/probe abasic site inhibits *Tth* endonuclease IV cleavage by preventing duplex DNA structure formation, highlighting the utility of 3TEC-PCR for SNP detection. Additionally, this result indicates that the blocked 3TEC-PCR forward primer/probe requires hybridisation to an absolute complementary target match to enable cleavage, extension, and signal production. This 3TEC-PCR property ensures increased assay specificity compared to standard allele-specific PCR methods by preventing the priming of partially homologous target sequences and reducing the occurrence of non-specific template-independent interactions such as primer-dimer formation. The synthetic DNA templates, SNP0 and SNP1, tested at 10^4^ copies to highlight 3TEC-PCR single-base specificity (Figure 3), produced similar amplification curves and Ct values compared to the *H. influenzae* genomic DNA tested at 10^4^ genome copies (Figure 1).

Multiplex 3TEC-PCR real-time target detection was successfully exhibited using the *H. influenzae*, *N. meningitidis,* and *S. pneumoniae* 3TEC-PCR multiplex assay (Figure 4). All three bacterial targets, tested at 10^4^ genome copies, were each detected in separate target reactions and in one combined reaction with all three targets, producing similar approximate Ct values in both formats: 26 for *H. influenzae*, 28 for *N. meningitidis,* and 29 for *S. pneumoniae*. However, it can be observed that there is a reduction in the fluorescence production between the single-target reactions and the combined multiple-target reaction. This is a result of the expected reaction inhibition from the simultaneous co-amplification of three bacterial targets in one reaction. Although bacterial pathogen co-infection can be rare, particularly with leading meningitis-associated organisms [33], this demonstration of pathogen co-amplification in a single reaction highlights the robustness of the 3TEC-PCR method. There is a slight reduction in single-target detection efficiency between the singleplex and multiplex 3TEC-PCR assays. The singleplex *H. influenzae* 3TEC-PCR wild-type assay produced at Ct value of 23.5 for *H. influenzae* at 10^4^ copies (Figure 2), compared to a Ct value of 26 in the multiplex assay (Figure 4). This reduced efficiency of target detection is a result of reaction inhibition due to the presence of additional primer sets in the multiplex reaction and is an expected limitation of multiplex PCR compared to singleplex PCR [34,35]. In terms of assay sensitivity, initial LOD analysis for the *H. influenzae*, *N. meningitidis*, and *S. pneumoniae* 3TEC-PCR multiplex assay demonstrated low-level detection of 10 genome copies per reaction for each target when tested separately. Multiplex PCR typically requires assay optimisation involving biasing primer sets based on assay performance to ensure that each set produces similar Ct values and satisfactory LODs [34]. The multiplex 3TEC-PCR assay in this study used unbiased, balanced primer set concentrations, without assay optimisation. However, resulting Ct values between each target were relatively similar, with low limits of detection observed, highlighting the compatibility of 3TEC-PCR with multiplexing applications.

Numerous PCR technologies with two or three-oligonucleotide systems facilitate real-time multiplex detection and single-base specificity; however, these approaches have various limitations compared to 3TEC-PCR. Three-oligonucleotide PCR systems incorporating forward and reverse primers flanking fluorescently labelled probes, such as hydrolysis probes, fluorescence resonance energy transfer hybridisation probes, or molecular beacons, enable multiplex detection and SNP identification using LNA modifications [12,27]. However, the design of three-oligonucleotide PCR systems can be restrictive when targeting biomarkers with limited suitable target sequence regions. Two-oligonucleotide PCR systems using novel primer/probe modifications with fluorescent labels, such as Scorpion [36], Amplifluor [37], LUX [38], Cyclicon [39], Angler [40], and Plexor [41], also enable multiplex detection and SNP identification. These methods, however, involve complicated probe design and are subject to the same previously outlined limitations for SNP detection as standard allele-specific PCR, with some approaches requiring additional nucleic acid analogues to enable single-base specificity [14]. Dual priming oligonucleotide (DPO) technology is a two-oligonucleotide PCR system that facilitates SNP detection via internal primer polydeoxyinosine linkers; however, for multiplex detection this method requires the incorporation of fluorescently labelled hydrolysis probes [42]. RNase H2-dependant PCR (rhPCR) is another two-oligonucleotide real-time PCR system that enables SNP detection using blocked primers with RNA residues that are cleaved upon target hybridisation by a thermostable RNase H2 enzyme [43]. The rhPCR method, however, has only been demonstrated in a singleplex format, with real-time multiplex detection requiring the inclusion of hydrolysis probes. Cycling probe technology (CPT) is a pre-cursor to rhPCR, with initial isothermal diagnostic applications involving RNase H cleavage of chimeric DNA-RNA-DNA probes [44], and subsequent applications utilising LNA hydrolysis probes for SNP detection [45]. However, the universal application of diagnostic methods utilising RNase H enzymes with RNA residue primers or probes is limited as the occurrence of non-specific cleavage by RNases other than RNase H, or inhibition due to human genomic DNA, has been reported [46]. Cairns and colleagues previously reported a two-oligonucleotide real-time PCR system using an enzyme-cleavable primer/probe with a 5′-end restriction enzyme sequence flanked by a fluorophore and quencher [47]. This method enables multiplex detection; however, as the restriction enzyme sequence is not target-specific, and due to reverse-primer activity, this system is subject to the same SNP detection limitations as standard allele-specific PCR.

3TEC-PCR technology, derived from our previously reported isothermal diagnostic methods incorporating cleavable primer/probe systems [48,49], requires minor alterations to standard real-time PCR assay design and performance. In addition to the inclusion of a modified primer/probe and *Tth* endonuclease IV, zinc chloride is also required as a cleavage enzyme stabilising agent. Primer/probe Tm values should be compatible with the 3TEC-PCR 65 °C annealing temperature, the optimal incubation temperate for *Tth* endonuclease IV. The thermostability of *Tth* endonuclease IV in combination with the blocked primer/probe facilitates hot-start PCR reactions. Placement of the primer/probe abasic site can be altered; however, the cleaved oligonucleotide should be of a sufficient Tm value to enable efficient post-cleavage primer activity. Alternate positioning of the fluorophore and quencher labels can be used, and cytosine residues as well as thymine residues can be utilised for internal oligonucleotide labelling. Positioning of the primer/probe quencher label is ideally located on a guanine residue to enable increased quenching activity [50]; however, this is not essential. Compared to standard three-oligonucleotide hydrolysis probe PCR systems, 3TEC-PCR does not need to accommodate an oligonucleotide probe between the forward and reverse primers, enabling improved variability of reverse primer positioning, increased design flexibility especially when targeting restrictive diagnostic biomarkers, and reduced assay oligonucleotide requirements. This study details the first report of a two-oligonucleotide real-time multiplex PCR method with single-base specificity using *Tth* endonuclease IV, providing effective transferable diagnostics technology for infectious disease pathogen detection and SNP identification.

## 4. Materials and Methods

### 4.1. Bacterial Strains, DNA Extraction, and Quantification

The bacterial reference strains used in this study included type strain *H. influenzae* DSM 4690, a range of *H. influenzae* capsular subtypes (a–f), and closely related *Haemophilus* strains, with *N. meningitidis* NCTC 10025 and *S. pneumoniae* DSM 20566 type strains (Table S1). Haemophilus test media (Oxoid, Hampshire, UK) was used to culture all *Haemophilus* strains, and brain heart infusion media (Oxoid) was used to culture the *N. meningitidis* and *S. pneumoniae* type strains. Bacterial incubation was carried out at 37 °C for 18 h under microaerophilic conditions. The DNeasy Blood and Tissue kit (Qiagen, Hilden, Germany) and Qubit dsDNA broad range kit (Life Technologies, Warrington, UK) were used as per manufacture instructions to extract and quantify DNA from all cultured strains. All bacterial strains and extracted DNA were stored at −80 °C prior to use. Genome copy number values for DNA extracted from each strain were generated using resulting DNA concentrations with approximate genome size standards of 1.83 Mb, 2.2 Mb and 2.1 Mb for *Haemophilus*, *N. meningitidis,* and *S. pneumoniae* strains, respectively.

### 4.2. Diagnostic Targets, Oligonucleotides, and Synthetic Templates

The *H. influenzae*, *N. meningitidis,* and *S. pneumoniae* diagnostic targets used for 3TEC-PCR assay design were the *fucK* gene (accession number L42023.1), the *NMO_1242* ABC transporter gene (accession number AM889136.1), and the *lepA* gene (accession number HE983624.1), respectively. All oligonucleotides (Table 1) containing modifications were synthesised using HPLC purification by Metabion International AG (Planegg, Germany), oligonucleotides without modifications were synthesised using standard desalting by Integrated DNA Technologies (Leuven, Belgium). Each forward primer/probe contained a 1′2′-dideoxyribose spacer modification in place of the nucleotide located 7 bases from the oligonucleotide 3′ end. The primer/probe fluorophores used, Cy5, FAM, and HEX, each corresponded to one of three fluorescence detection channels in the LightCycler 480 Instrument II (Roche Diagnostics, Sussex, UK) used to perform all 3TEC-PCR reactions. The synthetic templates used to demonstrate 3TEC-PCR single-base specificity and SNP differentiation were two 500 bp DNA gBlocks Gene Fragments, SNP0 and SNP1 (Table S2), synthesised by Integrated DNA Technologies. The SNP0 template is an exact copy of a 500 bp region of the *H. influenzae fucK* gene diagnostic target and acted as a wild-type template. The SNP1 template contains the same nucleotide sequence as SNP0 with an SNP in the target region of the *H. influenzae* wild-type forward primer/probe (Table 2) and acted as a mutant allele template.

### 4.3. Singleplex Detection

Demonstration of 3TEC-PCR singleplex detection was performed using the *H. influenzae* 3TEC-PCR wild-type assay. This assay contained 1X LightCycler 480 Probes Master (Roche Diagnostics), 0.5 µM *H. influenzae* wild-type forward primer/probe, 0.5 µM *H. influenzae* reverse primer, 10 U *Tth* endonuclease IV (New England Biolabs, Hitchin, UK), 25 µM zinc chloride (Sigma-Aldrich, Darmstadt, Germany), 1 µL DNA template or molecular-grade water to act as a negative control reaction, and molecular-grade water to give a final reaction volume of 20 µL. The 3TEC-PCR reactions, performed on a LightCycler 480 Instrument II, included an initial template denature phase at 95 °C for 2 min, followed by 45 cycles of an anneal and denature phase at 65 °C for 90 s and 95 °C for 30 s, respectively. Single fluorescence acquisition measurements were recorded at each anneal step in the Cy5 fluorescence detection channel (646 to 662 nm). The recorded fluorescent signal generated amplification curves data with cycle threshold (Ct) values identifying the cycles at which reactions produced exponential signal-exceeding background fluorescence, indicating positive or negative reactions. For demonstration of singleplex detection, the *H. influenzae* 3TEC-PCR wild-type assay was challenged with type strain *H. influenzae* DSM 4690 genomic DNA at 10^4^, 10^3^, 10^2^, 10^1^, and 10^0^ genome copies per reaction, testing each concentration in duplicate (Figure 2). A negative control reaction incorporating molecular-grade water in place of bacterial template was carried out in parallel.

### 4.4. Analytical Specificity and Sensitivity

An inclusivity panel of 10 *H. influenzae* references strains and an exclusivity panel of 30 closely related *Haemophilus* reference strains, with *N. meningitidis* and *S. pneumoniae* type strains, were used to demonstrate the analytical specificity of the *H. influenzae* 3TEC-PCR wild-type assay. Purified genomic DNA from each of the specificity panel bacterial strains was tested at 10^4^ genome copy concentrations (Table S1).

Replicates of serially diluted type strain *H. influenzae* DSM 4690 purified DNA were used to establish the analytical sensitivity of the *H. influenzae* 3TEC-PCR wild-type assay. Genomic DNA template concentrations of 32, 16, 8, 4, 2, and 1 genome copies were tested in replicates of 12. Probit analysis was performed on the resulting data to determine the assay limit of detection (LOD) with 95% confidence (Table S3). Negative control reactions were performed in parallel to the above specificity and sensitivity testing.

### 4.5. Allele-Specific Detection

For a demonstration of 3TEC-PCR single-base specificity and SNP differentiation, the *H. influenzae* 3TEC-PCR wild-type assay and the *H. influenzae* 3TEC-PCR mutant allele assay were used. The mutant allele assay was performed as per the wild-type assay with the *H. influenzae* mutant allele forward primer/probe replacing the wild-type forward primer/probe. Both assays were challenged with the SNP0 and SNP1 synthetic DNA templates (Table S2) at 10^4^ copies per reaction, with negative control reactions performed in parallel. The resulting fluorescent signal was recorded with the LightCycler 480 Instrument II using the Cy5 (646 to 662 nm) and FAM (495 to 520 nm) fluorescence-detection channels (Figure 3).

### 4.6. Multiplex Detection

The 3TEC-PCR real-time simultaneous detection of multiple bacterial targets was exemplified using the *H. influenzae*, *N. meningitidis,* and *S. pneumoniae* 3TEC-PCR multiplex assay. This reaction was performed as per the *H. influenzae* 3TEC-PCR wild-type assay with the addition of *N. meningitidis* and *S. pneumoniae* 3TEC-PCR oligonucleotides (Table 1). Bacterial target DNA templates were each added at 1 µL volumes, with molecular-grade water used to give a final reaction volume of 20 µL. For a demonstration of single-target detection, the multiplex assay was challenged in separate reactions with *H. influenzae* DSM 4690, *N. meningitidis* NCTC 10025, and *S. pneumoniae* DSM 20566 purified DNA, each at a concentration of 10^4^ genome copies per reaction. In parallel, to demonstrate simultaneous multiple target detection, the multiplex assay was challenged with all three bacterial targets in a single reaction at 10^4^ genome copy concentrations. A negative control reaction without any bacterial template was carried out in parallel. The resulting fluorescent signal was recorded with the LightCycler 480 Instrument II using the Cy5 (646 to 662 nm), FAM (495 to 520 nm) and HEX (535 to 565 nm) fluorescence-detection channels. For correction of any possible channel-to-channel fluorescence cross-talk, a colour compensation file was generated and applied as per the LightCycler 480 Instrument II operator manual.

## Figures and Tables

**Table 1:** 3TEC-PCR oligonucleotides.

Type	Sequence (5′-3′)
***H. influenzae***	
Forward Primer/Probe (Wild-Type)	Cy5-TGCCGCAATGTTTACCTTTGCA(dSpacer)GCGTAG-BHQ2
Forward Primer/Probe (Mutant Allele)	FAM-TGCCGCAATGTTTACCTTTGCA(dSpacer)**C**CGTAG-BHQ1
Reverse Primer	CTTGCTGTGCCGCGTTTACATTTTCGTAA
***N. meningitidis***	
Forward Primer/Probe	FAM-TGTTGGTGGTGTCGCTGTTTGA(dSpacer)ATTGTG-BHQ1
Reverse Primer	TGTGCAAACAGATACGTCCGCAAACCGCC
***S. pneumoniae***	
Forward Primer/Probe	HEX-TTTGGTGGTCGATGCGGCTCAA(dSpacer)GAATTG-BHQ1
Reverse Primer	AAGCCAGATAAACGTTGGCAAGAGTTTGA

Cy5, cyanine fluorophore; dSpacer, 1′,2′-dideoxyribose; BHQ2, black hole quencher 2; FAM, 6-carboxyfluorescein fluorophore; bold and underline, single-base difference between wild-type and mutant allele primer/probe; BHQ1, black hole quencher 1; HEX, 6-hexachlorofluorescein fluorophore.

**Table 2:** *H. influenzae* 3TEC-PCR wild-type forward primer/probe sequence with complementary targets of the SNP0 and SNP1 synthetic templates.

Primer/Probe	TGCCGCAATGTTTACCTTTGCA-GCGTAG
	|||||||||||||||||||||||||||||
SNP0	ACGGCGTTACAAATGGAAACGTCCGCATC
SNP1	ACGGCGTTACAAATGGAAACGTC**G**GCATC

Bold and underline, single-base mismatch.

## Supplementary Materials

Supplementary materials can be found at https://www.mdpi.com/article/10.3390/ijms22116061/s1.
