# Supplementary material for: 3′ Tth Endonuclease Cleavage Polymerase Chain Reaction (3TEC-PCR) Technology for Single-Base-Specific Multiplex Pathogen Detection using a Two-Oligonucleotide System
Source: Int J Mol Sci. 2021 Jun 4;22(11):6061. doi: 10.3390/ijms22116061 (PMC8199996; doi:10.3390/ijms22116061)
Supplement: Supplementary file 1 [file ijms-22-06061-s001.zip › ijms-1243209-supplementary.pdf]

Supplemental Data

Table S1: *Haemophilus influenzae* 3TEC-PCR wild-type assay specificity panel

Organism	Strain	3TEC-PCR Result
**Inclusivity Panel**		
H. influenzae reference strains		
H. influenzae (b, Type Strain)	DSM 4690	+
H. influenzae (a)	NCTC 8465	+
H. influenzae (b)	DSM 10001	+
H. influenzae (b)	DSM 11969	+
H. influenzae (b)	DSM 11970	+
H. influenzae (b)	DSM 24049	+
H. influenzae (c)	NCTC 8469	+
H. influenzae (d)	DSM 11121	+
H. influenzae (e)	NCTC 8472	+
H. influenzae (f)	DSM 10000	+
**Exclusivity Panel**		
Haemophilus reference strains (excl. *H. influenzae*) with *N. meningitidis* and *S. pneumoniae* type strains		
H. actinomycetemcomitans	DSM 8324	-
H. actinomycetemcomitans	DSM 11122	-
H. aphrophilus	NCTC 11096	-
H. avium	DSM 18557	-
H. ducreyi	DSM 8925	-
H. ducreyi	NCTC 11479	-
H. equigenitalis	DSM 10668	-
H. felis	DSM 21192	-
H. haemoglobinophilus	DSM 21241	-
H. haemolyticus	CCUG 12834	-
H. haemolyticus	CCUG 15312	-
H. haemolyticus	CCUG 15642	-
H. haemolyticus	CCUG 24149	-
H. haemolyticus	CCUG 34110	-
H. haemolyticus	CCUG 36015	-
H. haemolyticus	CCUG 36016	-
H. haemolyticus	CDC-M19501	-
H. haemolyticus	CDC-M21127	-
H. haemolyticus	CDC-M21621	-
H. haemolyticus	NCTC 10839	-
H. paracuniculus	DSM 21452	-
H. paragallinarum	DSM 18554	-
H. parahaemolyticus	DSM 21417	-
H. parainfluenzae	DSM 8978	-
H. paraphrohaemolyticus	DSM 21451	-
H. parasuis	DSM 21448	-
H. pittmaniae	DSM 17240	-
H. pittmaniae	DSM 21203	-
H. segnis	NCTC 10977	-
H. somnus	CCUG 12839	-
N. meningitidis (type strain)	NCTC 10025	-
S. pneumoniae (type strain)	DSM 20566	-

DSM, Leibniz Institute DSMZ - German Collection of Microorganisms and Cell Cultures; NCTC, National Collection of Type Cultures; CCUG, Culture Collection, University of Gothenburg, Sweden; CDC, Centre for Disease Control; +, positive; -, negative.

**Table S2: Synthetic DNA gBlocks Gene Fragment templates**
**SNP0 (without SNP):**
500 bp sectional copy of the *H. influenzae fucK* gene diagnostic target
GTTTGCTACTCGTGGAGAAATTTATGTCAGCGCATTACAACATATGGCAAATAAACTCAAAA ACGGTTTATCCGTGCTACATCAAGTCAGTCAGTTCCAAGCTAAAAGCTTAATTTGTGTTGGG GGTGGTTCAAAAAATGTATTGTGGAATCAAATCCGTGCAAACACCTTAAATTTACCGATTGA TGTTGTGGACATTTCTGAAAGTACTGTACTTGGTGCCGCAATGTTTACCTTTGCAGGCGTAGG CATTTACGAAAATGTAAACGCGGCACAGCAAGCGATGCAACCAACAAGAAAACGAATTTA CCCAAACTAGAAGGAACAACAAAATGCTAAAAGGTATTCACCCAGCTCTTTCTCCTGAATT ACTAAAAACCCTCGCCGAAATGGGGCATGGCGATGAGATAGTTCTAGCTGACGCCCATTTT CCCGCACATTCACTACATAAAAATGTAATACGTGCAGATGGTATATCTATAGATATTTTACT TGAAGCA
**SNP1 (with SNP):**
SNP0 sequence with an SNP in the target region of the *H. influenzae* wild-type forward primer/probe
GTTTGCTACTCGTGGAGAAATTTATGTCAGCGCATTACAACATATGGCAAATAAACTCAAAA ACGGTTTATCCGTGCTACATCAAGTCAGTCAGTTCCAAGCTAAAAGCTTAATTTGTGTTGGG GGTGGTTCAAAAAATGTATTGTGGAATCAAATCCGTGCAAACACCTTAAATTTACCGATTGA TGTTGTGGACATTTCTGAAAGTACTGTACTTGGTGCCGCAATGTTTACCTTTGCAG**<u>C</u>**CGTAGG CATTTACGAAAATGTAAACGCGGCACAGCAAGCGATGCAACCAACAAGAAAACGAATTTA CCCAAACTAGAAGGAACAACAAAATGCTAAAAGGTATTCACCCAGCTCTTTCTCCTGAATT ACTAAAAACCCTCGCCGAAATGGGGCATGGCGATGAGATAGTTCTAGCTGACGCCCATTTT CCCGCACATTCACTACATAAAAATGTAATACGTGCAGATGGTATATCTATAGATATTTTACT TGAAGCA
Bold and underline, SNP.

Table S3: *H. influenzae* 3TEC-PCR wild-type assay limit of detection (LOD) probit analysis

Genome copy concentration	Replicates tested / Replicates detected
32	12 / 12
16	12 / 12
8	12 / 12
4	12 / 11
2	12 / 10
1	12 / 7
**LOD with 95% probability:** 4.1 genome copies per reaction	
